# Novel serotonin 5-HT_2A_ receptor antagonists derived from 4-phenylcyclohexane-5-spiro-and 5-methyl-5-phenyl-hydantoin, for use as potential antiplatelet agents

**DOI:** 10.1007/s43440-021-00284-6

**Published:** 2021-06-11

**Authors:** Anna Czopek, Monika Kubacka, Adam Bucki, Agata Siwek, Barbara Filipek, Maciej Pawłowski, Marcin Kołaczkowski

**Affiliations:** 1 Department of Medicinal Chemistry, Faculty of Pharmacy, Jagiellonian University Medical College, 9 Medyczna Street, 30-688 Kraków, Poland; 2 Department of Pharmacodynamics, Jagiellonian University Medical College, 9 Medyczna Street, 30-688 Kraków, Poland; 3 Department of Pharmacobiology, Faculty of Pharmacy, Jagiellonian University Medical College, Medyczna 9, 30-688 Krakow, Poland

**Keywords:** Imidazolidinine-2,4-dione, 5-HT_2A_ receptors, Hydantoin, Antiplatelet, Aggregation

## Abstract

**Background:**

Antiplatelet drugs have been used in the treatment of acute coronary syndromes and for the prevention of recurrent events. Unfortunately, many patients remain resistant to the available antiplatelet treatment. Therefore, there is a clinical need to synthesize novel antiplatelet agents, which would be associated with different pathways of platelet aggregation, to develop an alternative or additional treatment for resistant patients. Recent studies have revealed that 5-HT_2A_ receptor antagonists could constitute alternative antiplatelet therapy.

**Methods:**

Based on the structures of the conventional 5-HT_2A_ receptor ligands, two series of compounds with 4-phenylcyclohexane-5-spiro- or 5-methyl-5-phenyl-hydantoin core linked to various arylpiperazine moieties were synthesized and their affinity for 5-HT_2A_ receptor was assessed. Further, we evaluated their antagonistic potency at 5-HT_2A_ receptors using isolated rat aorta and cells expressing human 5-HT_2A_ receptors. Finally, we studied their anti-aggregation effect and compared it with ketanserin and sarpogrelate, the reference 5-HT_2A_ receptor antagonists. Moreover, the structure–activity relationships were studied following molecular docking to the 5-HT_2A_ receptor model.

**Results:**

Functional bioassays revealed some of the synthesized compounds to be moderate antagonists of 5-HT_2A_ receptors. Among them, **13**, 8-phenyl-3-(3-(4-phenylpiperazin-1-yl)propyl)-1,3-diazaspiro[4.5]decane-2,4-dione, inhibited collagen stimulated aggregation (IC_50_ = 27.3 μM) being more active than sarpogrelate (IC_50_ = 66.8 μM) and comparable with ketanserin (IC_50_ = 32.1 μM). Moreover, compounds 2–5, 9–11, 13, 14 inhibited 5-HT amplified, ADP- or collagen-induced aggregation.

**Conclusions:**

Our study confirmed that the 5-HT_2A_ antagonists effectively suppress platelet aggregation and remain an interesting option for the development of novel antiplatelet agents with an alternative mechanism of action.

**Supplementary Information:**

The online version contains supplementary material available at 10.1007/s43440-021-00284-6.

## Introduction

Antiplatelet drugs have long been used in the treatment of acute coronary syndromes and for the prevention of recurrent events. Large clinical trials have confirmed that antiplatelet agents such as clopidogrel and aspirin are capable of reducing the risk of myocardial infarction, stroke, or death [1, 2]. However, many patients treated with these agents still experience recurrent atherothrombotic events [3]. Moreover, some are resistant to aspirin [cyclooxygenase-1 (COX-1) inhibitor] or clopidogrel (P2Y_12_ receptor antagonist), even when they are used in combination, which increases their risk of further cardiovascular events [4–6]. Therefore, there is still a clinical need to synthesize novel antiplatelet agentsinvolving different pathways of platelet aggregation, as an alternative or additional treatment for resistant patients.

Serotonin (5-HT) is a platelet activator, stored in platelet-dense granules [5]. Among the seven classes of 5-HT receptors, only 5-HT_2A_ has been found in platelets. Upon platelet activation, 5-HT is released to plasma and, in an autocrine manner, promotes further platelet activation, acting at 5-HT_2A_ receptors. Released serotonin is also a potent vasoconstrictor of coronary arteries with a damaged endothelium [5, 7, 8]. Furthermore, coronary artery disease exerts a stimulating effect on platelets, triggering them to release serotonin [9]. Several studies on animal models and humans have shown that 5-HT_2A_ receptor antagonists can inhibit platelet aggregation [5, 8]. 5-HT may also be partially responsible for the increased residual platelet reactivity observed in patients who are on clopidogrel treatment after coronary stent placement, while 5-HT_2A_ antagonists reduce high on-treatment platelet reactivity. Therefore, in addition to the established therapies, serotonin antagonism at 5-HT_2A_ receptors might be a promising approach to improve the treatment outcomes of patients with coronary artery disease [5, 10].

**Fig. 1:**
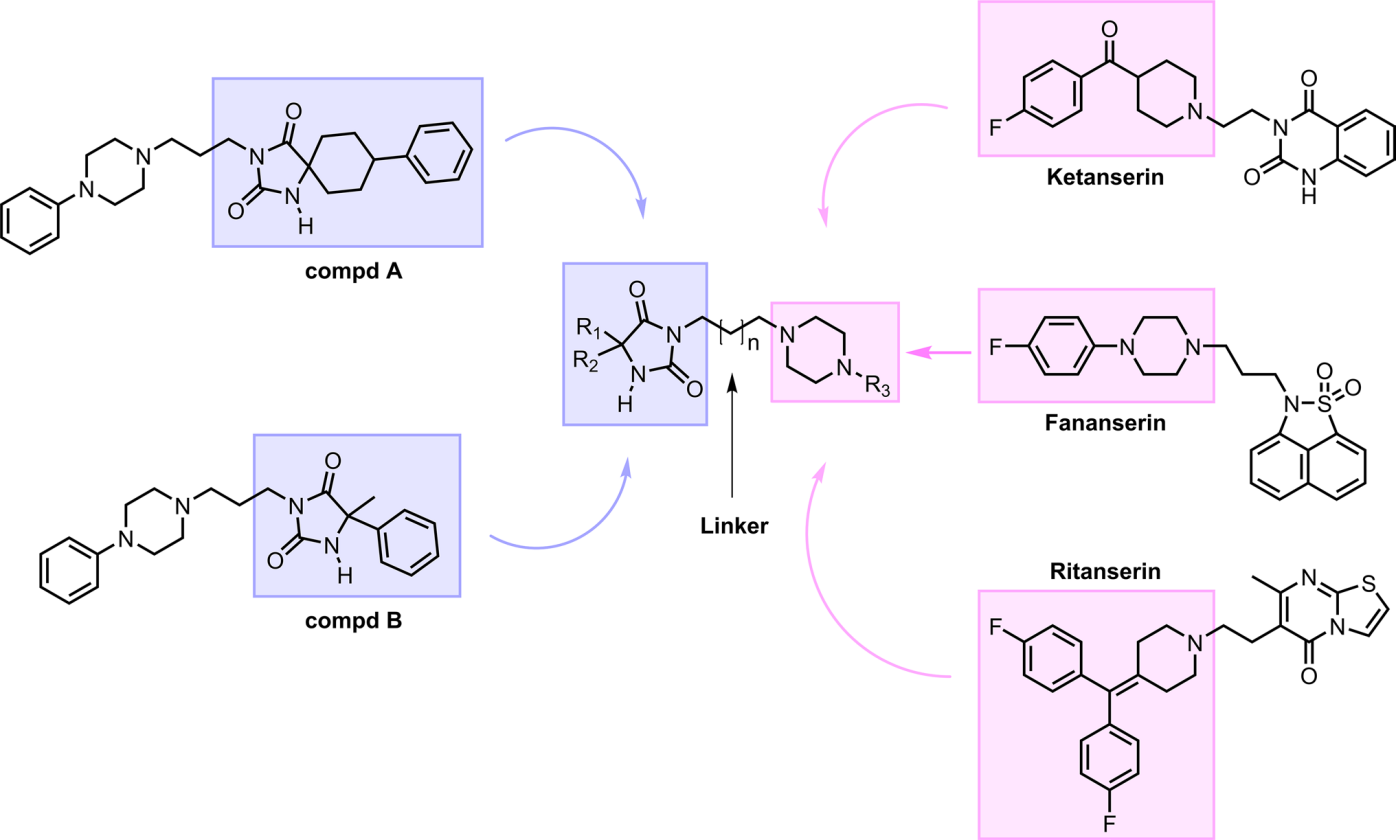
The general structures of designed compounds based on structure compounds **9** and **12** as well as conventional 5-HT_2A_ antagonists: ketanserin, fananserin and ritanserin

Our previous studies [11–13] showed that a differently substituted hydantoin ring connected to arylpiperazine moiety being the amine part, may interact with 5-HT_2A_ receptors and exert an antagonistic effect. Among these structures, two hydantoin derivatives with the highest affinity (K_i_ = 50 and 69 nM, respectively) and selectivity for 5-HT_2A_ receptors were selected, namely, 4-phenylcyclohexane-5-spirohydantoin (compound **A**) and 5-methyl-5-phenylhydantoin (compound **B**) linked with 4-phenypiperazin-1-yl-propyl moiety, which displayed significant antagonistic activity for 5-HT_2A_ receptors (Fig. 1). The compounds **A** and **B** fit the pharmacophore models of 5-HT_2A_ receptor antagonists proposed over the past two decades, which typically share two aryl rings or hydrophobic fragments and basic amine moiety separated from each other by a distance of 5.2–8.4 Å and 5.7–8.5 Å [14] (Supplementary material Figure S1). These results were a prerequisite for designing a new series of 5-methyl-5-phenylhydantoin and 4-phenylcyclohexane-5-spirohydantoin derivatives, which can serve as 5-HT_2A_ serotonin receptor ligands. The compounds have been designed as analogs of the aforementioned hydantoin derivatives (compound **A** and **B**) by combining structural fragments present in the conventional 5-HT_2A_ receptor antagonists, namely ketanserin (5-HT_2A_: K_i_ = 2 nM), ritanserin (5-HT_2A_: K_i_ = 0.45 nM), and fananserin (5-HT_2A_: K_i_ = 0.37 nM). The structural modifications carried out to develop novel 5-HT_2A_ receptor antagonists were (1) the extension of the alkyl spacer linking hydantoin with arylpiperazine moiety and (2) the introduction of a differently substituted arylpiperazine fragment in the amine part.

This manuscript describes the synthesis of the designed compounds and their affinity for the 5-HT_2A_ receptors. The intrinsic activity of the most promising compounds was evaluated, and molecular modeling studies were undertaken to identify the structural fragments responsible for their in vitro affinity. Finally, the anti-aggregation effect was tested after inducing the aggregation of whole rat blood with collagen and with 5-HT and ADP or collagen at the sub-threshold concentration.

## Materials and methods

### Chemistry

All chemicals and solvents were purchased from commercial suppliers (Aldrich and Chempur) and were used without further purification. Melting points were measured in open capillaries on an Electrothermal 9300 apparatus. Thin-layer chromatography (TLC) was run on Merck silica gel 60 F_254_ aluminium sheets (Merck; Germany), using the following solvents (*v:v*): (S_1_) dichloromethane (9)/methanol (0.3), (S_2_) ethyl acetate (9)/methanol (1), (S_3_) chloroform (7)/n-hexane (2)/acetone (2). Analytical HPLC was conducted on a Waters HPLC instrument with Waters 485 Tunable Absorbance Detector UV, equipped with a Symmetry column (C18, 3.5 µm, 4.6 × 30 mm) using water/acetonitrile gradient with 0.1% TFA as mobile phase at a flow rate of 5 ml/min. Additionally, the liquid chromatography/mass spectrometry (LC/MS) analysis was performed on the Waters Acquity TQD system, with a Waters TQD quadrupole mass spectrometer with detection by UV (DAD) using an Acquity UPLC BEH C18 column (1.7 μm, 2.1 × 100 mm). Water/acetonitrile gradient with 0.1% TFA was used as a mobile phase at a flow rate of 0.3 ml/min. The UPLC/MS purity of the investigated compounds (**1–12**) ranged to be over 95%. Elemental analyses (C, H, and N) for final compounds were carried out by a micro method using the elemental Vario EI III Elemental analyzer (Hanau, Germany). NMR spectra were recorded on Varian Mercury 300 MHz spectrometer (Varian Inc., Palo Alto, CA, USA) using the solvent (CDCl_3_) signal as an internal standard; chemical shifts are expressed in parts per million (ppm). Signal multiplets are represented by the following abbreviations: s (singlet), brs (broad singlet), d (doublet), t (triplet), m (multiplet). The Discover model, CEM microwave reactor was used to carry out the pressurized reaction.

**Scheme 1:**
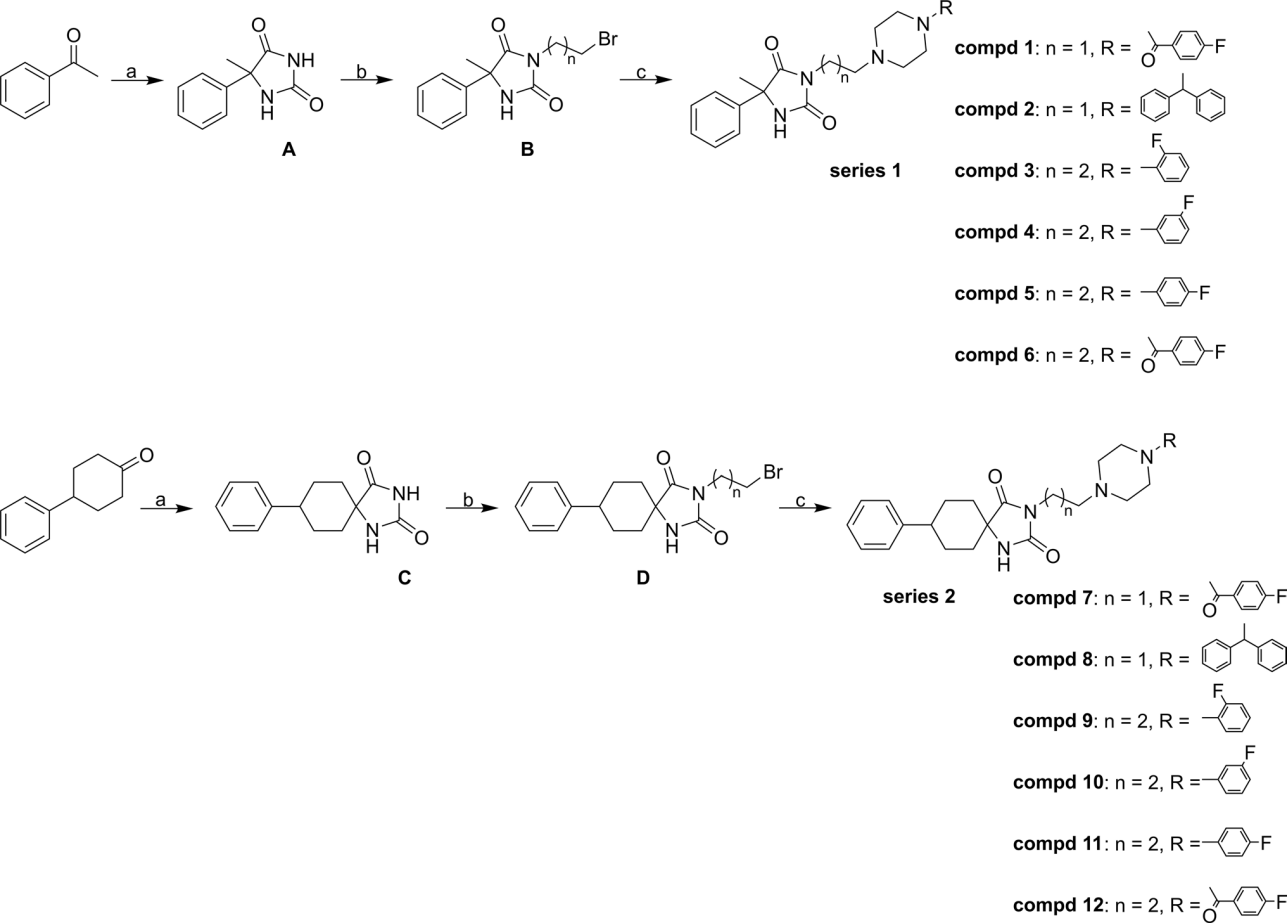
The synthetic routes of novel 5-methyl-5-phenylhydantoin (series 1) and 4-phenylcyclohexane-5-spirohydantoin (series 2) derivatives. Reagents and conditions: **A** KCN, (NH_2_)_2_CO_3_, 50% EtOH, 70 °C, 3 h; **B** Br(CH_2_)_n_Br, K_2_CO_3_, DMF, 70 °C, 3 h; **C** arylpiperazine, TEA, MeCN, MW: 200 W, 100 °C, 65–100 min (compd **1**–**6**, **8**, **9**, **11**)/arylpiperazine, K_2_CO_3_, 80˚C, MeCN, 24–48 h (compd **7**, **10**, **12**).

5-Methyl-5-phenylimidazolidine-2,4-dione (A, scheme 1), 8-phenyl-1,3-diazaspiro[4.5]decane-2,4-dione (C, scheme 1) and intermediate products (N3 halogealkyl derivatives of substituted imidazolidyno-2,4-diones) (B and D, scheme 1) [11, 12, 15] as well as (4-fluorophenyl)(piperazin-1-yl)methanone [16] were described earlier and the analytical results are consistent with those previously published.

#### General procedure for the synthesis of final compounds (1–12)

The final compounds (**1**–**12**) were obtained within two different synthetic pathways, described in procedure A and B. Procedure A (**1–6**, **8**, **9**, **11**): the final compounds were synthesized in a CEM microwave reactor (Discover model). The intermediate compound (0.3 mmol), trimethylamine (0.6 mmol) and appropriate piperazine derivatives (0.36 mmol) in acetonitrile (4 mL) was added to the microwave flask, and stirred for 65–100 min, at 100 °C, 200 W. Upon cooling to room temperature, the reaction mixture was concentrated in vacuum and purified by column chromatography. Procedure B (**7**, **10**, **12**): the reaction was carried out in a round bottom flask on a magnetic stirrer. The intermediate compound (0.33 mmol), appropriate piperazine derivatives (0.4 mmol), and potassium carbonate (0.66 mmol) in acetonitrile (10 mL) were heated for 24–48 h at 80 °C, with continuous stirring. After concentration in a vacuum, extraction was carried out with methylene chloride, the organic layer was dried, concentrated, and purified using column chromatography.

The analytical results of 8-phenyl-3-(3-(4-phenylpiperazin-1-yl)propyl)-1,3-diazaspiro[4.5]decane-2,4-dione (**13**) and 3-(3-(4-(3-chlorophenyl)piperazin-1-yl)propyl)-8-phenyl-1,3-diazaspiro[4.5]decane-2,4-dione (**14**) were previously described [11]. Detailed descriptions of compounds **1**, **6**, **7**, **8**, and **12** are in Supplementary materials.

##### 3-(2-(4-Benzhydrylpiperazin-1-yl)ethyl)-5-methyl-5-phenylimidazolidine-2,4-dione (2)

White powdery crystals. Yield: 72%; mp 212–215 °C; TLC: *R*_*f*_ = 0.31 (S_1_); HPLC: *t*_R_ = 1.491; MS calcd for [M + H]^+^: C_29_H_32_N_4_O_2_ *m/z*: 468.25, found: 469.27; ^1^H NMR (300 MHz, CDCl_3_-*d*) δ ppm 1.81 (s, 3 H, -C*H*_*3*_) 2.28 (br. s., 4 H, Pip) 2.47 (br. s., 4 H, Pip) 2.57 (t, *J* = 6.45 Hz, 2 H, -C*H*_*2*_-Pip) 3.61 (t, *J* = 6.45 Hz, 2 H, Hyd-C*H*_*2*_-) 4.13 [s, 1 H, -C*H*-(Ph)_2_] 7.13—7.20 (m, 2 H, Ph, Hyd) 7.22–7.30 (m, 5 H, Ph) 7.31–7.42 (m, 7 H, Ph) 7.47–7.54 (m, 2 H, Ph). Anal. calcd for C_29_H_32_N_4_O_2_ (468.60): C: 74.33, H: 6.88, N: 11.96; Found C: 74.08, H: 7.02, N: 11.50.

##### 3-(3-(4-(2-Fluorophenyl)piperazin-1-yl)propyl)-5-methyl-5-phenylimidazolidine-2,4-dione (3)

White powdery crystals. Yield: 83%; mp 126–129 °C; TLC: *R*_*f*_ = 0.25 (S_1_); HPLC: *t*_R_ = 1.217; MS calcd for [M + H]^+^: C_23_H_27_FN_4_O_2_ *m/z*: 410.21, found: 411.38; ^1^H NMR (300 MHz, CDCl_3_-*d*) δ ppm 1.77–1.88 (m, 5 H, -CH_3_, -CH_2_-C*H*_*2*_-CH_2_-) 2.39 (t, *J* = 7.03 Hz, 2 H, -C*H*_*2*_-Pip) 2.56 (t, *J* = 4.7 Hz, 4 H, Pip) 3.07 (t, *J* = 4.7, 5.3 Hz, 4 H, Pip) 3.54 (t, *J* = 7, 7.1 Hz, 2 H, Hyd-C*H*_*2*_-) 6.86–7.08 (m, 5 H, Ph) 7.28–7.42 (m, 3 H, Hyd, Ph) 7.47–7.54 (m, 2 *H,* Ph*)*. Anal. calcd for C_23_H_27_FN_4_O_2_ (410.49): C: 67.30, H: 6.63, N: 13.65; Found C: 66.95, H: 6.72, N: 13.19.

##### 3-(3-(4-(3-Fluorophenyl)piperazin-1-yl)propyl)-5-methyl-5-phenylimidazolidine-2,4-dione (4)

White powdery crystals. Yield: 33%; mp 125–126 °C; TLC: *R*_*f*_ = 0.26 (S_1_); HPLC: *t*_R_ = 1.253; MS calcd for [M + H]^+^: C_23_H_27_FN_4_O_2_ *m/z*: 410.21, found: 411.18; ^1^H NMR (300 MHz, CDCl_3_-*d*) δ ppm 1.77–1.89 (m, 5 H, -CH_2_-C*H*_*2*_-CH_2_-, -C*H*_3_) 2.38 (t, *J* = 7, 7.1 Hz, 2 H, -C*H*_*2*_-Pip) 2.47–2.55 (t, *J* = 4.7, 5.3 Hz, 4 H, Pip) 3.08–3.16 (t, *J* = 4.7, 5.3 Hz, 4 H, Pip) 3.58 (t, *J* = 7.33 Hz, 2 H, Hyd-C*H*_*2*_-) 6.46–6.67 (m, 4 H, Hyd, Ph) 7.11–7.22 (m, 1 H, Ph) 7.29–7.42 (m, 3 H, Ph) 7.47–7.53 (m, 2 H, Ph). Anal. calcd for C_23_H_27_FN_4_O_2_ x H_2_O (428.51): C: 64.47, H: 6.82, N: 13.08; Found C: 64.66, H: 6.55, N: 12.63.

##### 3-(3-(4-(4-Fluorophenyl)piperazin-1-yl)propyl)-5-methyl-5-phenylimidazolidine-2,4-dione (5)

White powdery crystals. Yield: 71%; mp 141–143 °C; TLC: *R*_*f*_ = 0.54 (S_2_); HPLC: *t*_R_ = 1.235; MS calcd for [M + H]^+^: C_23_H_27_FN_4_O_2_ *m/z*: 410.21, found: 411.25; ^1^H NMR (300 MHz, CDCl_3_-*d*) δ ppm 1.79–1.90 (m, 5 H, -CH_3,_ CH_2_-C*H*_*2*_-CH_2_-) 2.41 (t, *J* = 7.33 Hz, 2 H, -C*H*_*2*_-Pip) 2.56 (t, *J* = 4.1, 5.3 Hz, 4 H, Pip) 3.07 (t, *J* = 4.7, 5.3 Hz, 4 H, Pip) 3.58 (t, *J* = 7.03 Hz, 2 H, Hyd-C*H*_*2*_-) 6.35 (s, 1 H, Hyd) 6.79–6.88 (m, 2 H, Ph) 6.90–6.99 (m, 2 H, Ph) 7.29–7.42 (m, 3 H, Ph) 7.45–7.53 (m, 2 H, Ph). Anal. calcd for C_23_H_27_FN_4_O_2_ (410.49): C: 67.30, H: 6.63, N: 13.65; Found C: 66.82, H: 6.70, N: 13.36.

##### 3-(3-(4-(2-Fluorophenyl)piperazin-1-yl)propyl)-8-phenyl-1,3-diazaspiro[4.5]decane-2,4-dione (9)

White powdery crystals. Yield: 53%; mp 179–181 °C; TLC: *R*_*f*_ = 0.34 (S_1_); HPLC: *t*_R_ = 1.523; MS calcd for [M + H]^+^: C_27_H_33_FN_4_O_2_ *m/z*: 464.26, found: 465.28; ^1^H NMR (300 MHz, CDCl_3_-*d*) δ ppm 1.66–1.82 (m, 4 H, Cyclohexane) 1.75 (q, *J* = 2.9, 11.7, 11.8 Hz, 4 H, Cyclohexane) 1.85–2.14 (m, 7 H, Cyclohexane, -CH_2_-C*H*_*2*_-CH_2_-, Ph-C*H-*) 2.47 (t, *J* = 7.33 Hz, 2 H, -C*H*_*2*_-Pip) 2.61 (t, *J* = 4.1 Hz, 4 H, Pip) 3.08 (t, *J* = 4.1, 4.6 Hz, 4 H, Pip) 3.65 (t, *J* = 7.03 Hz, 2 H, Hyd-C*H*_*2*_-) 6.87–7.07 (m, 4 H, Ph) 7.19–7.25 (m, 1 H, Ph) 7.30–7.36 (m, 4 H, Ph) 8.06 (s, 1 H, Hyd). Anal. calcd for C_23_H_25_N_4_O_3_F (424.48): C: 69.80, H: 7.16, N: 12.06; Found C: 69.58, H: 7.22, N: 12.18.

##### 3-(3-(4-(3-Fluorophenyl)piperazin-1-yl)propyl)-8-phenyl-1,3-diazaspiro[4.5]decane-2,4-dione (10)

White powdery crystals. Yield: 52%; mp 193–195 °C; TLC: *R*_*f*_ = 0.37 (S_1_); HPLC: *t*_R_ = 1.546; MS calcd for [M + H]^+^: C_27_H_33_FN_4_O_2_ *m/z*: 464.26, found: 465.27; ^1^H NMR (300 MHz, CDCl_3_-*d*) δ ppm 1.74 (q, *J* = 3.5, 11.7, 11.8 Hz, 4 H, Cyclohexane) 1.86–2.09 (m, 7 H, Cyclohexane, -CH_2_-C*H*_*2*_-CH_2_-, Ph-C*H-*) 2.44 (t, *J* = 7.33 Hz, 2 H, -C*H*_*2*_-Pip) 2.55 (t, *J* = 4.7, 5.3 Hz, 4 H, Pip) 3.16 (t, *J* = 4.7, 5.3 Hz, 4 H, Pip) 3.65 (t, *J* = 7.03 Hz, 2 H, Hyd-C*H*_*2*_-) 6.47–6.66 (m, 3 H, Ph) 7.12–7.25 (m, 2 H, Ph) 7.30–7.35 (m, 4 H, Ph) 8.05 (s, 1 H, Hyd Anal. calcd for C_23_H_25_N_4_O_3_F (424.48): C: 69.80, H: 7.16, N: 12.06; Found C: 69.92, H: 7.34, N: 11.86.

##### 3-(3-(4-(4-Fluorophenyl)piperazin-1-yl)propyl)-8-phenyl-1,3-diazaspiro[4.5]decane-2,4-dione (11)

White powdery crystals. Yield: 60%; mp 216–217 °C; TLC: *R*_*f*_ = 0.31 (S_1_); HPLC: *t*_R_ = 1.526; MS calcd for [M + H]^+^: C_27_H_33_FN_4_O_2_ *m/z*: 464.26, found: 465.28; ^1^H NMR (300 MHz, CDCl_3_-*d*) δ ppm 1.70–1.84 (q, *J* = 9.4, 12.9, 14.6, 4 H, Cyclohexane) 1.85–2.13 (m, 7 H, Cyclohexane, -CH_2_-C*H*_*2*_-CH_2_-, Ph-C*H-*) 2.45 (t, *J* = 7.33 Hz, 2 H, -C*H*_*2*_-Pip) 2.56 (t, *J* = 4.7 Hz, 4 H, Pip) 3.07 (t, *J* = 4.7 Hz, 4 H, Pip) 3.65 (t, *J* = 7.03 Hz, 2 H, Hyd-C*H*_*2*_-) 6.78—6.87 (m, 2 H, Ph) 6.88–6.99 (m, 2 H, Ph) 7.18–7.25 (m, 1 H, Ph) 7.28–7.39 (m, 4 H, Ph) 8.38 (s, 1 H, Hyd). Anal. calcd for C_23_H_25_N_4_O_3_F (424.48): C: 69.80, H: 7.16, N: 12.06; Found C: 69.87, H: 7.32, N: 11.81.

### Radioligand binding assay

#### Preparation of solutions of test and reference compounds

Stock solutions of tested compounds (10 mM) were prepared in DMSO. Serial dilutions of compounds were prepared in 96-well microplate in assay buffers using an automated pipetting system epMotion 5070 (Eppendorf). Each compound was tested in eight concentrations from 10^–5^ to 10^–12^ M (final concentration).

#### 5-HT_2A_ receptor binding assay

Radioligand binding was performed using membranes from CHO-K1 cells stably transfected with the human 5-HT_2A_ receptor (PerkinElmer). All assays were carried out in duplicates. Data were fitted to a one-site curve-fitting equation with Prism 6 (GraphPad Software) and K_i_ values were estimated from the Cheng − Prusoff equation. The full description of this assay is in Supplementary material.

### Functional bioassays

#### In vitro functional bioassays at cells transfected with human 5HT_2A_ receptor (aequorin and luminescence-based intracellular calcium assay)

Tested and reference compounds were dissolved in DMSO at a concentration of 10^–2^ M. Serial dilutions were prepared in 96-well microplate in assay buffer and eight to ten concentrations were tested.

A cellular aequorin-based functional assay was performed with recombinant Chinese hamster ovary cells expressing mitochondrially targeted aequorin, human G-protein-coupled receptors, and the promiscuous G protein α16 for 5-HT_2A_. The full description of this assay is in Supplementary materials.

#### Functional bioassays at 5HT_2A_-receptors in isolated rat aorta

Isolated rat aorta was used to determine the antagonistic activity of studied compounds for 5HT_2A_-receptors. The male Wistar rats weighting 200–350 g were anesthetized with thiopental sodium (75 mg/kg i.p.) and the aorta was dissected and placed in a Krebs–Henseleit solution (NaCl 118 mM, KCl 4.7 mM, CaCl_2_ 2.25 mM, MgSO_4_ 1.64 mM, KH_2_PO_4_ 1.18 mM, NaHCO_3_ 24.88 mM, glucose 10 mM, C_3_H_3_O_3_Na 2.2 mM, EDTA 0.05 mM), denuded of the endothelium, cleaned of surrounding fat tissues and cut into 4-mm-long rings. The aortic rings were incubated in 30 ml chambers filled with a Krebs–Henseleit solution at 37 °C and pH 7.4 with constant oxygenation (O_2_/CO_2_, 19:1). Two stainless steel stirrups were inserted through the lumen of each aortic segment: one stirrup was attached to the bottom of the chamber and the other to an isometric FDT10-A force–displacement transducer (BIOPAC Systems, Inc., COMMAT Ltd., Turkey). The aortic rings were stretched and maintained at optimal tension of 2 g and allowed to equilibrate for 2 h.

After the equilibration period, the aortic rings were contracted to maximal tension with KCl (60 mM). The depolarizing solution KCl (60 mM) was obtained by equimolar substitution of NaCl for KCl. Then the cumulative concentration–response curves to 5-HT were determined in the absence and presence of antagonist. Tissues were incubated with antagonists for 30 min.

Concentration–response curves were analyzed using GraphPad Prism 6.0 software (GraphPad Software Inc., San Diego, CA, USA). Contractile responses to 5-HT (in the presence or absence of tested compounds) are expressed as a percentage of the maximal KCl effect reached in the concentration–response curves obtained before incubation with the tested compounds (Emax = 100%). Data are the mean ± SEM of three separate experiments. The affinity was estimated with the equation pK_B_ = log(concentration ratio–1)-log(molar antagonist concentration), where the concentration ratio is the ratio of equi-effective agonist concentrations in the absence and in the presence of the antagonist.

#### In vitro whole blood aggregation tests

**Fig. 2:**
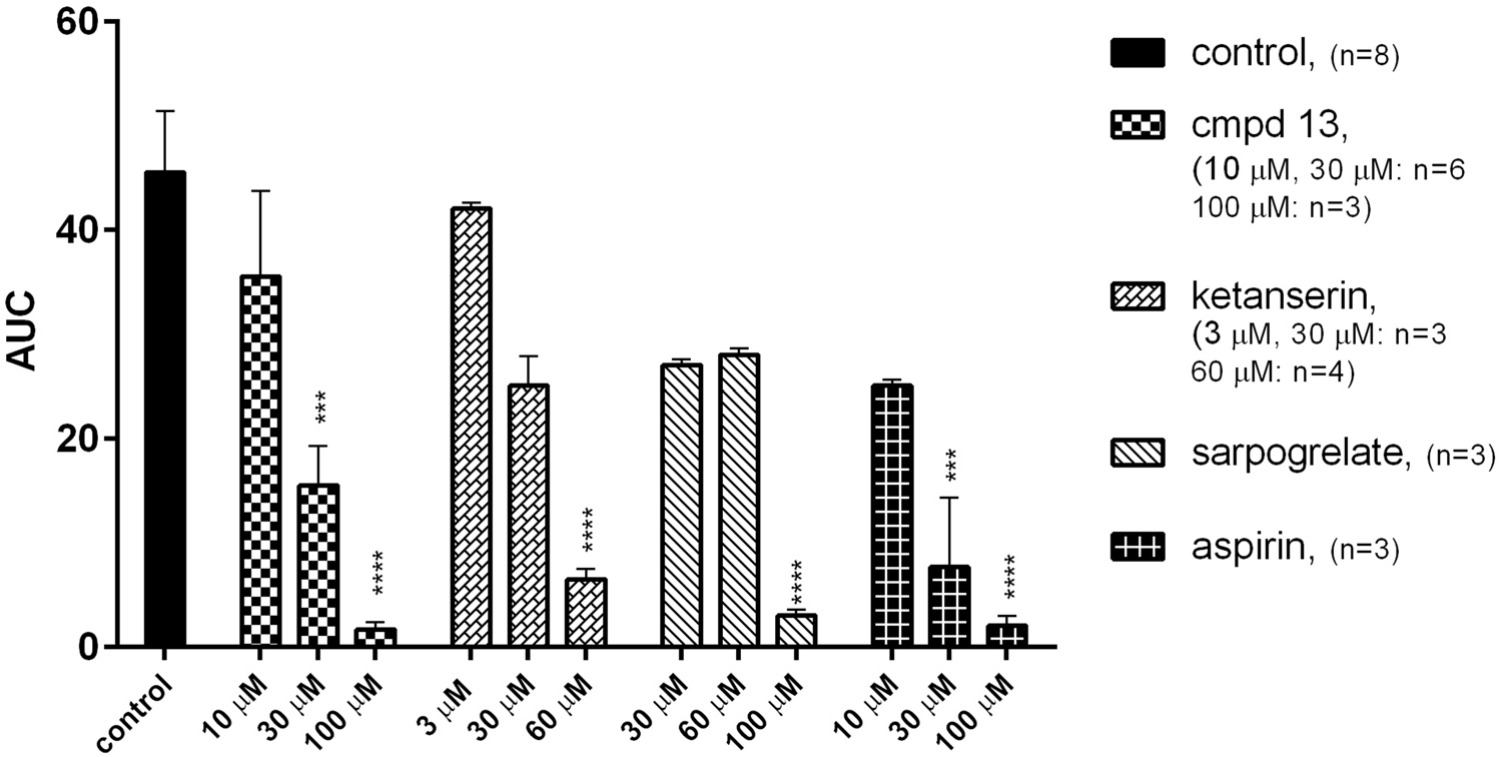
Effects of compound **13**, sarpogrelate, ketanserin and aspirin on in vitro whole rat blood aggregation induced by collagen (1.6 μg/ml) Results are expressed as mean ± SEM, ***p < 0.001, ****p < 0.0001 versus control group (0.1% DMSO in saline), one-way ANOVA, post hoc Dunnet test. *AUC* area under curve

**Fig. 3:**
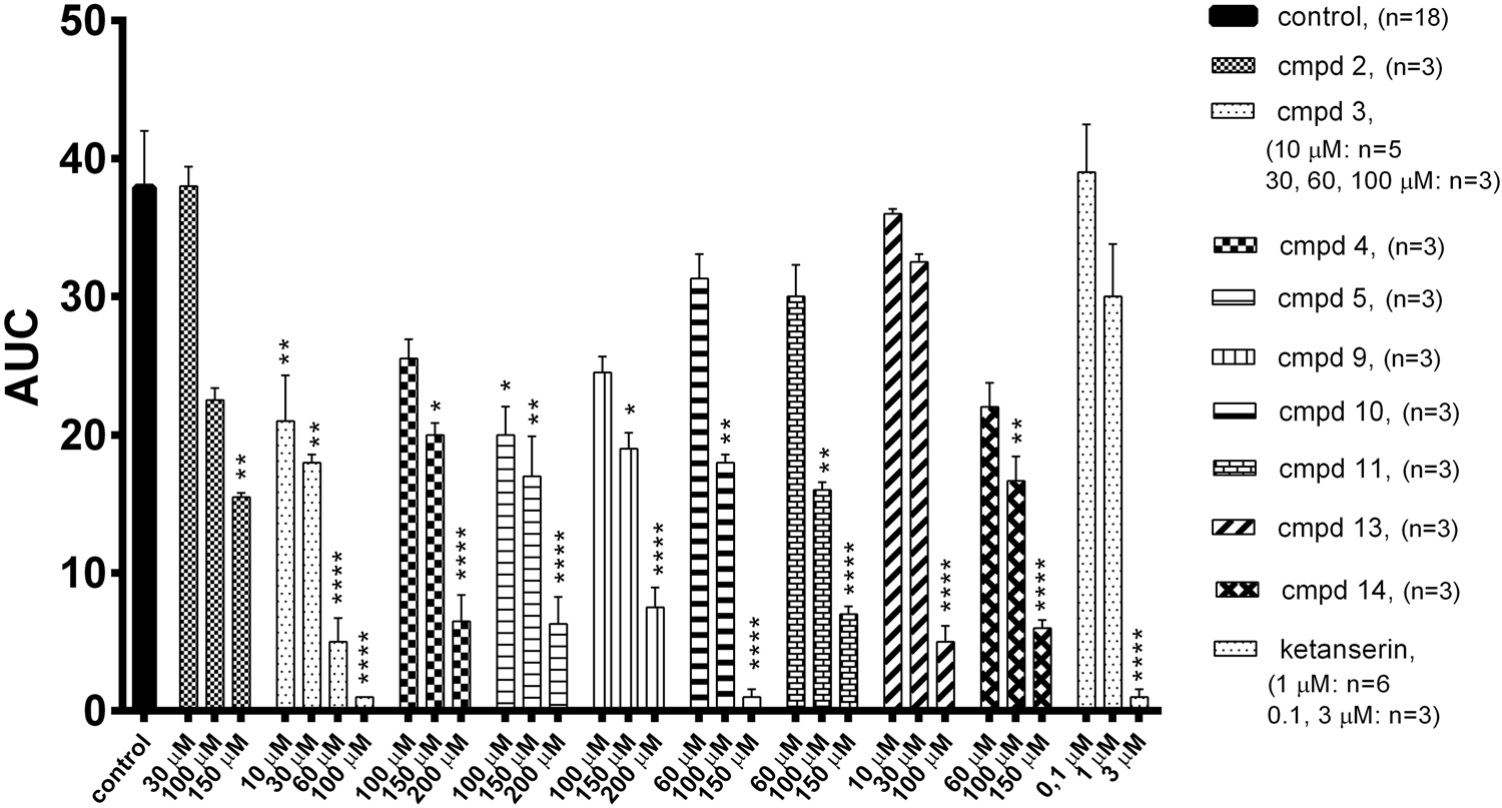
Effects of compounds **2**, **3**, **4**, **5**, **9**, **10**, **11**, **13**, **14** and ketanserin on in vitro whole rat blood aggregation induced by simultaneous addition of 5-HT (30 μM) and collagen (0.8 μg/ml). Results are expressed as mean ± SEM, *p < 0.05, **p < 0.01, ****p < 0.0001 versus control group (0.1% DMSO in saline), one-way ANOVA, post hoc Dunnet test. *AUC* area under curve

**Fig. 4:**
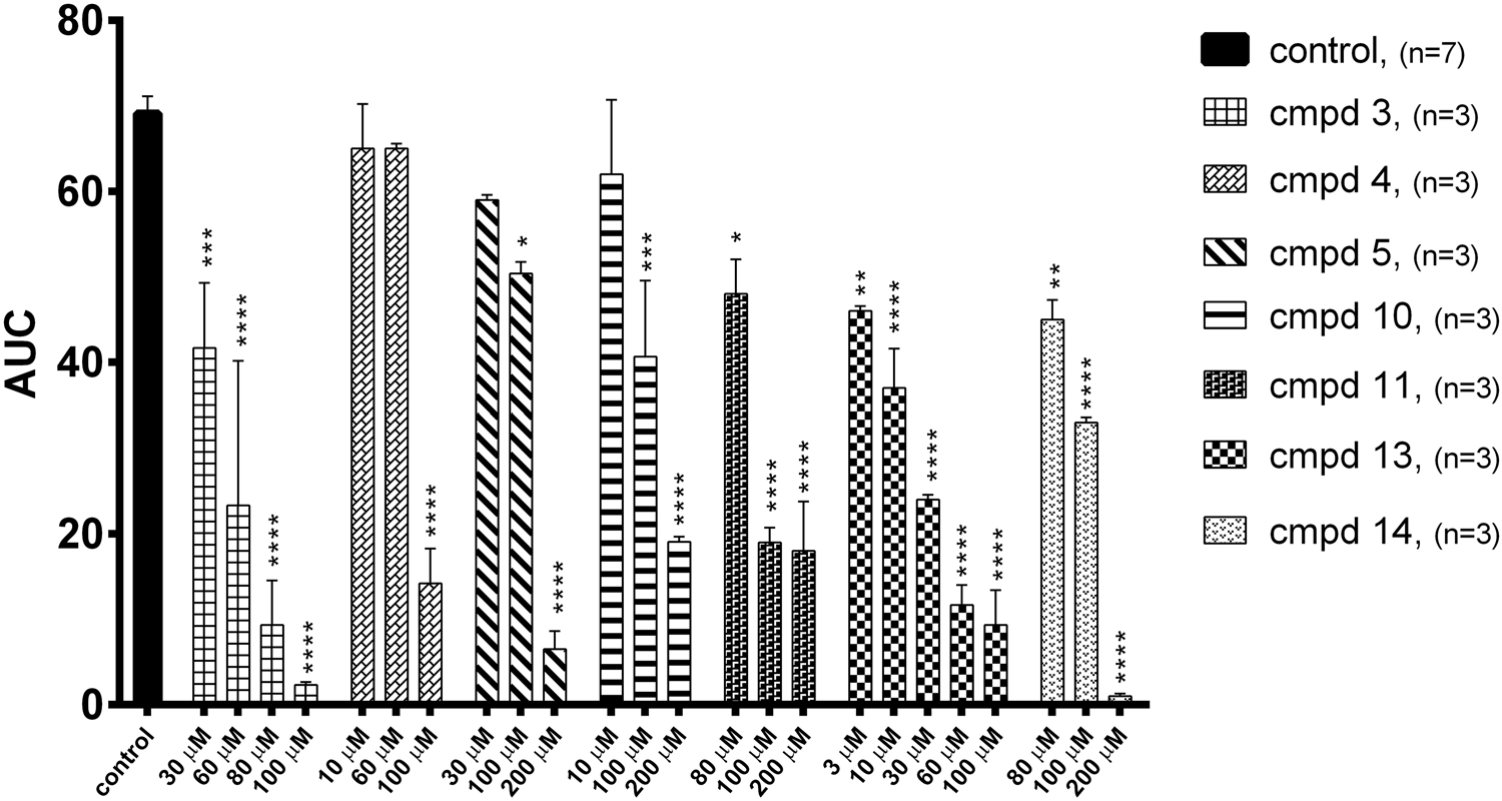
Effects of compounds **3**, **4**, **5**, **10**, **11**, **13**, **14** on in vitro whole rat blood aggregation induced by simultaneous addition of 5-HT (6 μM) and ADP (1.6 μM). Results are expressed as mean ± SEM, *p < 0.05, **p < 0.01, ***p < 0.001, ****p < 0.0001 versus control group (0.1% DMSO in saline), one-way ANOVA, post hoc Dunnet test. *AUC* area under curve

In vitro aggregation assays were performed using freshly drawn whole rat blood with a Multiplate platelet function analyzer (Roche Diagnostic), the five-channel aggregometer based on measurements of electric impedance. Blood was collected from carotid arteries with a hirudin blood tube (Roche Diagnostic). 300 μl of hirudin anticoagulated blood was mixed with 300 μl prewarmed isotonic saline solution containing studied compound or vehicle (deionized water or DMSO 0.1%) and preincubated for 3 min at 37 °C with continuous stirring. Aggregation was induced by adding collagen (Hyphen-Biomed, France) (final concentration 1.6 µg/ml), 5-HT (30 µM) and sub-threshold concentration of collagen (0.8 µg/ml), 5-HT (6 µM) and sub-threshold concentration of ADP (ADP test, Roche Diagnostic), (1.6 µM). The aggregation process was recorded for 6 min. Ketanserin (ketanserin ( +) tartrate salt, Sigma-Aldrich, Germany), sarpogrelate (sarpogrelate hydrochloride, Sigma-Aldrich, Germany), and aspirin (Tocris, UK) were used as reference compounds. The Multiplate software analyzed the area under the curve (AUC) of the clotting process of each measurement and calculated the mean values. Each concentration of studied compounds was tested at least three times. The exact sample size (n value) is presented on Figs. 2–4. Concentration-inhibition curves were constructed and analyzed by non-linear curve fitting using GraphPad Prism 6.0 (GraphPad Software Inc., San Diego, CA, USA).

#### Statistical analysis

Data were presented as mean ± standard error the mean (SEM). Statistical comparisons were made by the one-way analysis of variance (one-way ANOVA) and the significance of the differences between the control group and treated groups was determined by the Dunnet post hoc test. *p* < 0.05 was considered significant.

### Molecular modeling studies

Molecular docking was performed to the previously developed homology models of the 5-HT_2A_ receptor. The procedure for obtaining ligand-optimized models of high predictive value was characterized in detail previously [17, 18]. The 5-HT_2A_ receptor model was based on the 5-HT_2B_ receptor crystal structure 4IB4 [19]. Glide XP flexible docking was carried out using the OPLS3 force field and default parameters. H-bond constraint, as well as centroid of a grid box for docking to the receptor models were located on Asp86^3.32^. The selection of the poses was based on the prevalence of well-scored (glide gscore) complexes and visual evaluation of binding interactions. Ligand structures were optimized using LigPrep. The presented computational tools were implemented in Small-Molecule Drug Discovery Suite (Schrodinger, Inc.), which was licensed for Jagiellonian University Medical College.

### In silico toxicity prediction

In silico toxicity was assessed with a novel approach (pkCSM) which uses graph-based signatures to develop predictive models of ADMET properties or toxicity of introduced structures [20].

## Results and discussion

### Chemistry

The final compounds (series 1 and 2) were synthesized as outlined in Scheme 1. The substituted imidazolidine-2,4-dione derivatives (A and C) used as starting compounds were prepared from 1-phenylethanone or 4-phenylcyclohexanone, respectively, by the Bucherer–Bergs reaction followed by N3-alkylation with dihalogenoalkanes in the hydantoin ring. The resulting intermediates (B and D) were coupled with appropriate arylpiperazines in the presence of potassium carbonate or triethylamine to obtain the final compounds. The crude final products were purified using column chromatography. The structure and purity of the synthesized compounds were confirmed by spectral and chromatographic analyses, and the reaction progress was monitored by thin-layer chromatography. The detailed physicochemical and spectral data are summarized in the Experimental Part.

### In vitro 5-HT_2A_ receptor activity of compound 1–14

The novel series of compounds targeting serotonin 5-HT_2A_ receptors were designed by coupling the amide part of 4-phenylcyclohexane-5-spiro- or 5-methyl-5-phenylimidazolidine-2,4-dione with various arylpiperazine moieties, using linkers of different lengths. The heterocyclic imide ring, or substituted imidazolidine-2,4-dione in this case, seemed to be a good starting point for designing 5-HT_2A_ receptor ligands because it is also present in the structures of the conventional 5-HT_2A_ receptor antagonists such as ketanserin and fananserin (Fig. 1). In the amine part, the piperazine analogs (4-fluorophenyl)(piperidin-4-yl)methanone and 4-(bis(4-fluorophenyl)methylene)-piperidine, found in ketanserin and ritanserin, were introduced. Furthermore, a key structural fragment of fananserin, 4-fluorophenyl-1-piperazine, was chosen as the amine part, as well as its analogs with a fluorine atom at positions 2 and 3.

In the synthesized series, ten compounds (**2**–**6**, **9**–**14**) displayed low-to-high binding affinity for the 5-HT_2A_ receptors (K_i_ = 21–2584 nM, Table 1). Compounds containing phenyl(piperazin-1-yl)methanone (**1**, **6**, **7**, **12**) or 1-(diphenylmethyl)piperazine (**2**) fragment (except compound **2**) showed low or no affinity, while compound **2**, which is a 1-(diphenylmethyl)piperazine derivative with 5-methyl-5-phenylimidazolidine-2,4-dione moiety, showed a moderate affinity for the tested serotonin receptors (K_i_ = 450 nM, Table 1). However, the replacement of the aforementioned piperazines by fluorophenylpiperazine led to an increase in their affinity. Interestingly, compounds **5** and **11** containing a 4-fluorophenyl-1-piperazine fragment, which is present in fananserin, as well as compounds **13** and **14** having 1-phenylpiperazine and 3-chlorophenyl-1-piperazine moiety, respectively, displayed the highest affinity for the 5-HT_2A_ receptors in the prepared series (K_i_ = 21–50 nM). Already published compounds **13** and **14** showed selectivity for serotonin 5-HT_1A_ and dopamine D_2_ receptors [11] (for details see Supplementary materials Table S1). Moreover, the position of the fluorine atom in the 1-phenylpiperazine fragment seemed to determine the affinity of compounds for 5-HT_2A_ receptors as shifting the fluorine atom of this fragment from position 4 to position 3 or 2 caused a significant (6- to 10-fold) decrease in affinity (Table 1). The influence of the linker length on the compounds’ affinity for 5-HT_2A_ receptors was also investigated. It was observed that compounds with a linker having two methylene groups showed no affinity for the receptors (except compound **2**), but the elongation of the linker to three methylene groups resulted in an increased binding affinity.

**Table 1:** The radioligand binding data of 4-phenylcyclohexane-5-spirohydantoin and 5-methyl-5-phenylhydantoin derivatives and reference drug (mianserin) for serotonin 5-HT_2A_ receptors

	Compd	*n*	R	K_i_ [nM] ± SEM
	**1**	0	–CO4FPh	nd
	**2**	0	–CH(Ph)_2_	450.0 ± 43.9
	**3**	1	–2FPh	516.0 ± 52.0
	**4**	1	–3FPh	340.0 ± 57.0
	**5**	1	–4FPh	48.0 ± 4.9
	**6**	1	–CO4FPh	nd
	**7**	0	–CO4FPh	nd
	**8**	0	–CH(Ph)_2_	nd
	**9**	1	–2FPh	220.0 ± 19.6
	**10**	1	–3FPh	297.0 ± 19.9
	**11**	1	–4FPh	21.0 ± 1.7
	**12**	1	–CO4FPh	2584.0 ± 302.0
	**13 (Compd A)**^**a,b**^	1	–Ph	20.0 ± 4.8^a^
	**14**^a^	1	–3ClPh	24.0 ± 2.5^a^
	Mianserin			1.2 ± 0.2

The data for **13**, **14**, were already published in [11] and re-printed in the current manuscript for comparison, with permission from Acta Poloniae Pharmaceutica—Drug Research

*nd* no affinity detected

^a^The data for **13**, **14**, were already published in [11] and re-printed in the current manuscript for comparison, with permission from Acta Poloniae Pharmaceutica—Drug Research

^b^Compd A Fig. 1

For further characterization of the compounds, K_i_ (5-HT_2A_) < 1000 nM was set as selection criteria. Based on this, compounds **2**–**5**, **9**–**11** were chosen for further determination of functional activity for the 5-HT_2A_ receptors. The binding affinity of compounds **13** and **14** has been studied and published in our previous work [11].

### Biofunctional assays

The antagonistic activity of compounds **2**–**5**, **9**–**11** for 5-HT_2A_ receptors was analyzed by evaluating the inhibition of serotonin-induced contraction in rat aorta. The tested compounds shifted the 5-HT concentration–response curve to the right without lowering the maximal response, which indicated that they are competitive antagonists of the rat 5-HT_2A_ receptors (Figure S2). The affinities of these compounds were expressed as pK_B_ values, estimated from the 5-HT concentration–response curves and a single concentration of the compounds. The pK_B_ values ranged from 6.161 (compound **2**) to 7.430(compound **11**) (Table 2) and were in line with the radioligand binding data. Additionally, compounds **2**–**5**, **9**, and **11** were studied in cells transfected with human 5-HT_2A_ receptors. The results of this bioassay revealed that compounds **3** and **11** are moderate antagonists of the human 5-HT_2A_ receptors (pK_B_ ≈ 6.758, Table 2). The antagonistic activity of compounds **13** and **14** has been studied and published in our previous work [11].

**Table 2:** In vitro functional antagonist data of tested 4-phenylcyclohexane-5-spirohydantoin and 5-methyl-5- phenylhydantoin derivatives and reference drugs (ketanserin, sapogrelate) for 5-HT_2A_ receptors [a] in rat aorta and [b] in cells expressing human 5-HT_2A_ receptors

Compd	pK_B_ ± SEM	
	a	b
**2**	6.161 ± 0.06	5.706 ± 0.21
**3**	6.666 ± 0.12	6.758 ± 0.15
**4**	6.357 ± 0.18	5.035 ± 0.23
**5**	6.957 ± 0.23	5.032 ± 0.30
**9**	7.018 ± 0.08	5.702 ± 0.16
**10**	6.300 ± 0.36	nt
**11**	7.430 ± 0.32	6.757 ± 0.06
**13 (Compd A)**	7.665 ± 0.03^a^	nt
**14**	7.110 ± 0.05^a^	nt
Ketanserin	9.439 ± 0.09^b^	7.839 ± 0.03^b^
Sarpogrelate	8.220 ± 0.08^b^	7.296 ± 0.04^b^

*nt* not tested

^a,b^The data for ^a^**13**, **14**, as well as for ^b^ketanserin and sarpogrelate were already published in [7, 11], respectively, and re-printed in the current manuscript for comparison, with permission from European Journal of Pharmacology and Acta Poloniae Pharmaceutica—Drug Research

### In vitro whole blood aggregation tests

Compounds with confirmed 5-HT_2A_ antagonistic activity (**2**–**5**, **9**–**11**, **13**, **14**) were subjected to further studies. First, to evaluate the antiplatelet effect, freshly isolated rat whole blood was incubated with the selected compounds (3–200 μM) or vehicle (DMSO), and the aggregation responses were recorded. Platelet aggregation was triggered by collagen or using collagen or ADP at the subthreshold concentration and 5-HT. Ketanserin and sarpogrelate, which exert antiplatelet activity due to 5-HT_2A_ antagonism as well as aspirin (COX-1 inhibitor) were used as reference compounds for this analysis.

When studying aggregation, various agonists can be used individually to identify a pathway that may be potentially affected by a test compound. Collagen is one of the most important, natural platelet agonist. The final effect of collagen stimulation is increased intracellular Ca^2+^ concentration, which leads to morphological changes, ADP and 5-HT secretion, and the synthesis of thromboxane A2 (TXA2) [21]. Therefore using collagen as an aggregation agonist, provides a general means of assessing platelet function and anti-aggregation effect of test compounds in vitro [21]. Figure 2 shows the effect of compound **13**, ketanserin, sarpogrelate, and aspirin on aggregation induced by collagen. In our study, only aspirin and the most potent 5-HT_2A_ receptor antagonists were able to inhibit collagen-induced aggregation. These compounds significantly decreased platelet aggregation (*F*_12,38_ = 9.873, *p* < 0.0001). Among the obtained compounds, only compound **13,** which was identified as the most potent 5-HT_2A_ antagonist, was able to inhibit collagen-stimulated aggregation of whole rat blood (IC_50_ = 27.3 ± 3.8 μM). Its anti-aggregation effect was greater than that of sarpogrelate (IC_50_ = 66.8 ± 12.9 μM) and was comparable to ketanserin (IC_50_ = 32.1 ± 3.0 μM). For comparison, the IC_50_ value for aspirin was equal to 14.5 ± 1.1 μM (Table 3, Fig. 2).

**Table 3:** Potencies of studied and reference compounds in inhibiting whole rat blood aggregation in vitro induced by [a] collagen (1.6 μg/ml), [b] 5-HT (30 μM) and collagen (0.8 μg/ml), [c] 5-HT (6 μM) and ADP (1.6 μM)

Compound	a (collagen) IC_50_ [μM]	b (5-HT + collagen) IC_50_ [μM]	c (5-HT + ADP) IC_50_ [μM]
2	na	139.8 ± 18.9	na
3	na	17.4 ± 10.9	37.9 ± 6.5
4	na	153.1 ± 26.4	83.1 ± 8.1
5	na	127.2 ± 32.7	123.7 ± 36.2
9	na	138.6 ± 16.1	na
10	na	94.3 ± 53.5	118.6 ± 32.1
11	na	91.3 ± 7.1	140.5 ± 44.6
13 (Compd A)	27.3 ± 3.8	53.3 ± 4.6	9.9 ± 2.5
14	na	75.8 ± 16.6	93.8 ± 9.4
Sarpogrelate	66.8 ± 12.9	nt	22.7 ± 2.1^a^
Ketanserin	32.1 ± 3.0	1.3 ± 0.0	9.7 ± 2.1^a^
Aspirin	14.5 ± 1.1	nt	nt

*na* not active, *nt* not tested

^a^The data for ketanserin and sarpogrelate were already published in [7] and re-printed in the current manuscript for comparison, with permission from European Journal of Pharmacology

Further, the antiplatelet activity was studied using 5-HT as a co-agonist of aggregation induced by collagen or ADP at the sub-threshold concentration. Physiologically, many factors that induce platelet aggregation act synergistically. 5-HT itself is only a weak platelet activator, but it can enhance the aggregation process induced by collagen or ADP, thus activating platelet 5-HT_2A_ receptors [5, 22, 23]. Collagen used at a concentration of 0.8 μg/ml did not induce aggregation of whole rat blood, while 5-HT used alone could not cause aggregation at any concentration tested. However, the combination of 5-HT at a concentration of 30 μM with collagen at the subliminal concentration resulted in the maximal aggregation response (Fig. 3). This aggregation effect was attenuated by the studied compounds (**2**–**5**, **9**–**11**, **13**, **14**) and ketanserin [(*F*_31,84_ = 7.808, *p* < 0.0001)], (Fig. 3). The obtained IC_50_ values ranged from 17.4 ± 10.9 μM (compound **3**) to 153.1 ± 26.4 μM (compound **4**), (Table 3). The reference compound ketanserin inhibited blood aggregation with an IC_50_ value of 1.3 ± 0.01 μM, which confirmed its high antagonistic activity for 5-HT_2A_ receptors found in functional studies.

The tested compounds also inhibited 5-HT-amplified and ADP-induced platelet aggregation. At a concentration of 1.6 μM, ADP used alone could cause only partial and transient aggregation of rat blood in vitro, while 5-HT used alone did not induce aggregation at any concentration tested. However, combining 5-HT at a concentration of 6 μM with ADP at the sub-threshold concentration resulted in a maximal aggregation response. The serotonin-mediated amplification of ADP-stimulated aggregation was attenuated when rat blood was preincubated with compounds **3**, **4**, **5**, **10**, **13**, and **14** (*F*_24,54_ = 20.60, *p* < 0.0001), with the IC_50_ values ranging from 9.9 ± 2.5 μM (compound **13**) to 140.5 ± 44.6 μM (compound **11**). For comparison, the IC_50_ value of ketanserin was similar to that of compound **13** and was equal to 9.7 ± 2.1 μM [7]. As observed for induction with collagen alone, compound **13** was more potent than sarpogrelate, which IC_50_ value, found in our previous work was equal to 22.7 ± 2.1 [7]. On the other hand, even at a concentration of 200 μM, compound **2** did not exhibit any significant inhibitory effect on ADP- and 5-HT-induced blood aggregation, which proved its lowest intrinsic activity toward 5-HT_2A_ receptors found in the biofunctional study. Besides compound **13**, compound **3** was observed to be the most potent in the entire series of newly synthesized compounds, with an IC_50_ value of 17.4 ± 10.9 μM against collagen- and 5-HT-induced blood aggregation and a value of 37.9 ± 6.5 μM against ADP- and 5-HT induced blood aggregation. Thus, the effect of compound **3** on ADP- and 5-HT-induced aggregation was comparable to that of sarpogrelate. The results of this analysis are presented in Fig. 4 and Table 3.

It was found that the most potent 5-HT_2A_ receptor antagonists—compound **13**, ketanserin, and sarpogrelate—inhibited collagen-induced platelet aggregation, whereas other 5-HT_2A_ antagonists—compounds **2**–**5**, **9**–**11**, and **14**—inhibited 5-HT-potentiated platelet aggregation. Among these, compound **3** was the most active and inhibited collagen- or ADP-induced and 5-HT-amplified aggregation with the lowest IC_50_ values.

### Molecular modeling studies

**Fig. 5:**
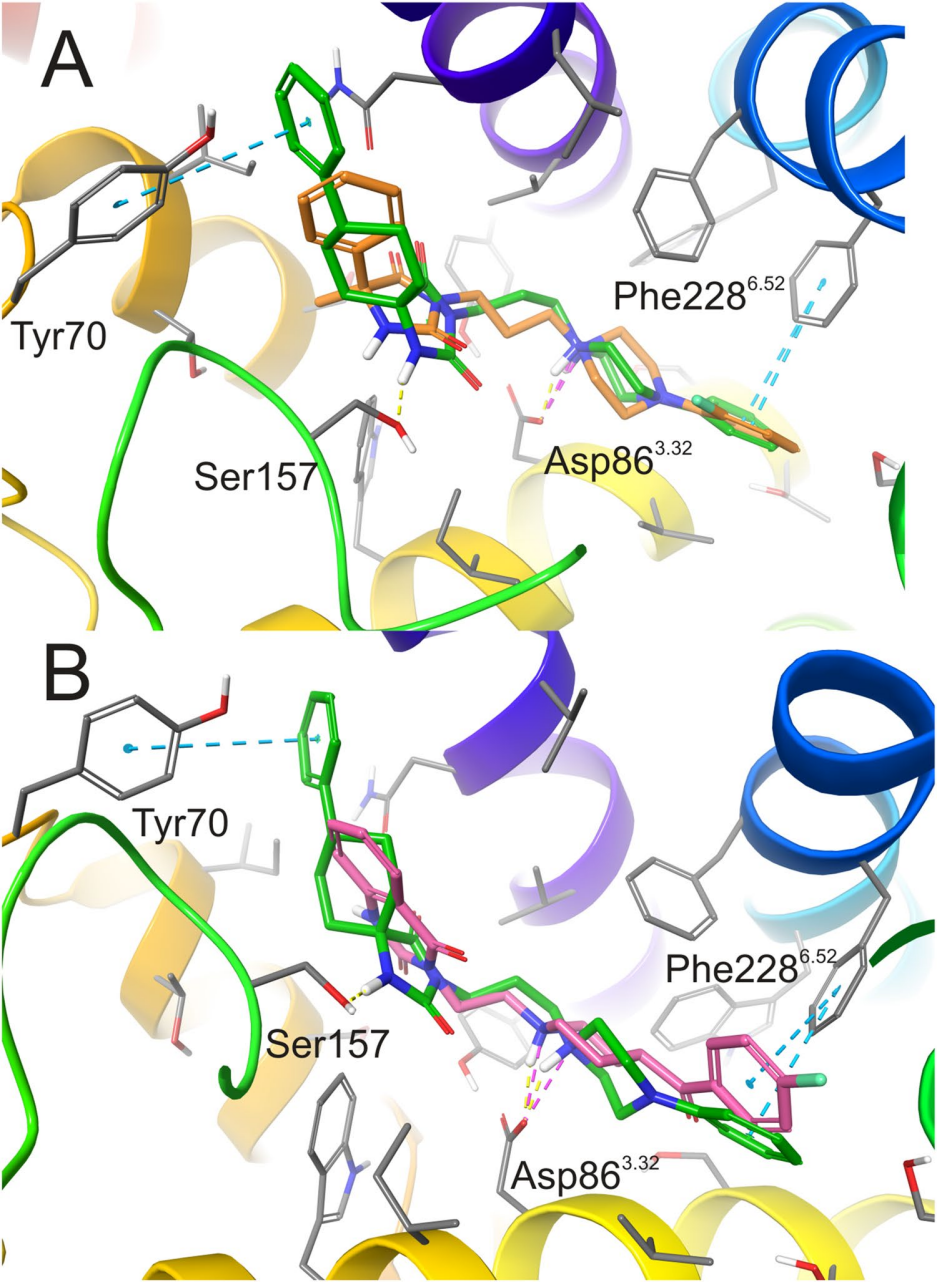
The predicted binding mode of compound **13** (green) displayed together with (A) compound **3** (orange) and (B) reference compound ketanserin (pink) in the 5-HT_2A_ receptor homology model based on 4IB4. The arylpiperazine moieties interact with Asp86^3.32^ (salt bridge/charge-reinforced hydrogen bond) and Phe228^6.52^ (CH-*π* stacking) while hydantoin-containing fragment of compound **13** interacts exclusively with Tyr70 (*π*-*π* stacking) and Ser157 (H-bond) from extracellular loops 1 and 2, respectively. Amino acid residues engaged in ligand binding (within 4 Å from the ligand atoms) are represented as sticks

The lead compounds **3** and **13**, although having different arylpiperazine substitutions, both showed a significant affinity for 5-HT_2A_ receptors in the radioligand binding assays. Nevertheless, compared to compound **3**, compound **13** showed over 25-fold higher affinity for the target protein. To analyze this difference at the molecular level, docking to the homology model was performed. In the case of both compounds, the arylpiperazine moiety bound in the orthosteric binding site, as expected for the monoaminergic ligands. This moiety was anchored through the salt bridge (charge-assisted hydrogen bond) between the basic nitrogen atom and Asp86^3.32^, as well as through CH–π stacking between the aromatic ring and Phe228^6.52^ (Fig. 5A). The hydantoin-containing fragments of the compounds were found to be located in the accessory cavity of the receptor. In the case of compound **13** (but not compound 3), the spiro-cyclohexane structure in this region provided a favorable conformation, allowing the formation of *π*–*π* interactions between the phenyl ring and Tyr70. Moreover, the hydantoin itself formed an H-bond with Ser157 and overlapped with 1,2,3,4-tetrahydroquinazoline-2,4-dione moiety of the reference 5-HT_2A_ receptor ligand ketanserin (Fig. 5B). The above relationships may help in the design of further 5-HT_2A_ receptor ligands of the proposed chemotype.

### In silico toxicity prediction

In preliminary studies, the balance between activity, potency, pharmacokinetics, and toxicity is crucial for effective drug candidates. Therefore, computational approaches to optimize pharmacokinetics and toxicity properties have been developed that allow the initial estimation of these parameters for the newly synthesized structures. One of these predictive models was used to assess the *in silico* toxicity of the most active compounds **2**–**5**, **9**–**11**, **13**, and **14** (for detailed information please see Table S2 in the Supplementary materials) with a hydantoin core.

Based on the prediction results, the tested compounds showed no toxicity in the AMES test, which measures the potential mutagenic activity. Moreover, one of the hERG predictive models indicated no cardiotoxicity of the tested compounds, while the other suggested a possible interaction with the potassium channel. Besides, this model predicted the possibility of hepatotoxicity of some compounds, but these results require further evaluation in in vitro tests.

## Conclusion

A series of compounds with 5-methyl-5-phenylhydantoin and 4-phenylcyclohexane-5-spirohydantoin core connected to arylpiperazine moiety via a methylene linker were designed and synthesized (**1**–**14**) as potential 5-HT_2A_ receptor antagonists. The analysis of binding affinity and functional bioassays showed that a majority of these compounds exhibited moderate affinity and antagonistic activity for rat and human serotonin 5-HT_2A_ receptors, which further led to the evaluation of their anti-aggregation effect. In the entire series, compound **13** was found to be the most potent, as it inhibited collagen-stimulated aggregation of whole rat blood. The anti-aggregation effect of compound **13** was comparable to that of ketanserin and greater than sarpogrelate. The tested compounds were also analyzed for their inhibitory effect on 5-HT-amplified, and ADP- or collagen-induced platelet aggregation. The 5-HT-dependent aggregation studies showed compounds **3** and **13** as the most active. From the results of this study, it can be concluded that 5-HT_2A_ antagonists may effectively inhibit platelet aggregation and are an interesting target for the development of novel antiplatelet agents with an alternative mechanism of action.

## Supplementary Information

Below is the link to the electronic supplementary material.

Supplementary file1 (PNG 351 KB)

Supplementary file2 (JPG 981 KB)

Supplementary file3 (DOCX 518 KB)
