# Supplementary figures and images for: Novel serotonin 5-HT2A receptor antagonists derived from 4-phenylcyclohexane-5-spiro-and 5-methyl-5-phenyl-hydantoin, for use as potential antiplatelet agents
Source: Pharmacol Rep. 2021 Jun 11;73(5):1361–72. doi: 10.1007/s43440-021-00284-6 (PMC8460535; doi:10.1007/s43440-021-00284-6)

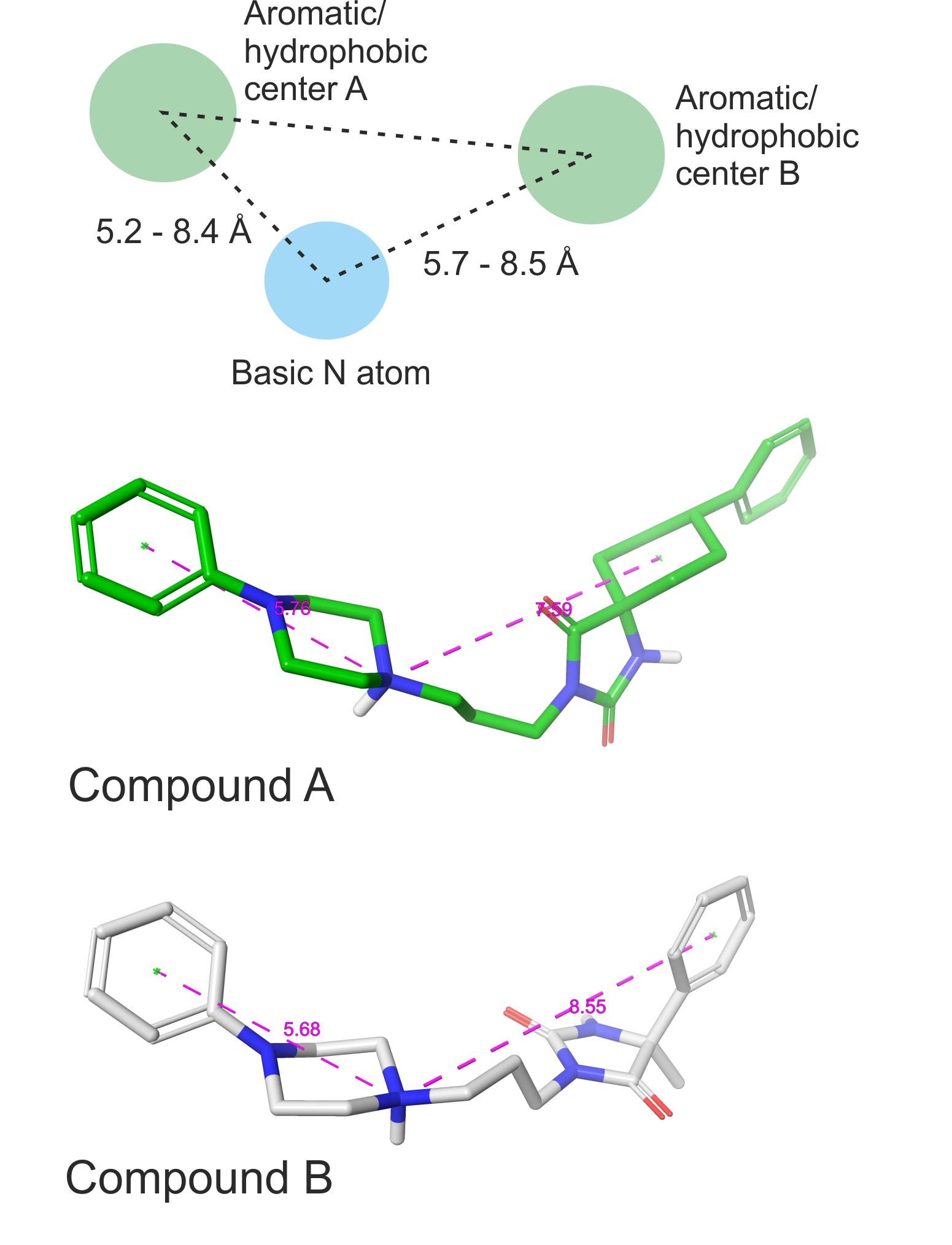

Supplement: Supplementary file 1 — Supplementary file1 (PNG 351 KB) [file 43440_2021_284_MOESM1_ESM.png]

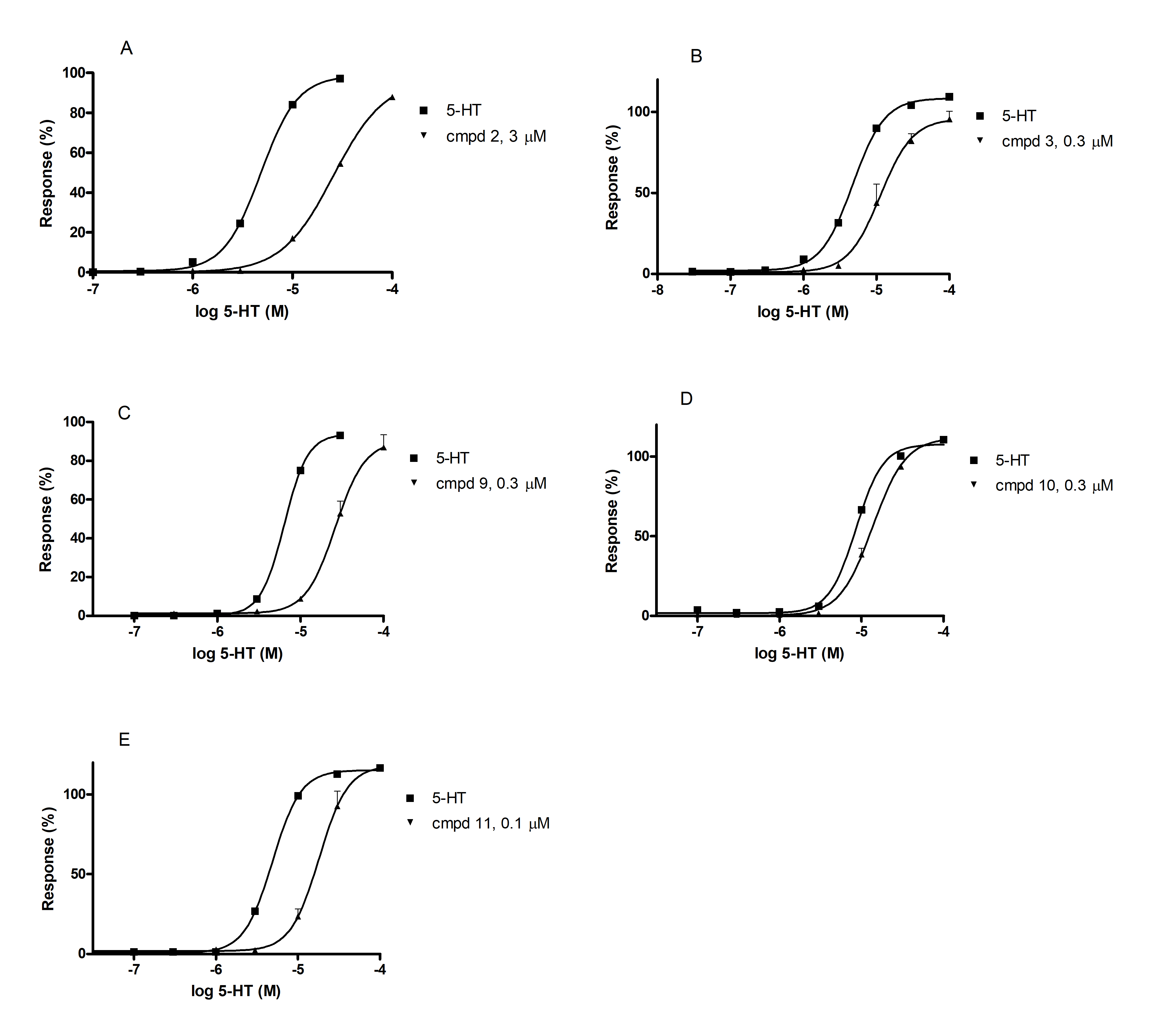

Supplement: Supplementary file 2 — Supplementary file2 (JPG 981 KB) [file 43440_2021_284_MOESM2_ESM.jpg]
