# Supplementary material for: Novel serotonin 5-HT2A receptor antagonists derived from 4-phenylcyclohexane-5-spiro-and 5-methyl-5-phenyl-hydantoin, for use as potential antiplatelet agents
Source: Pharmacol Rep. 2021 Jun 11;73(5):1361–72. doi: 10.1007/s43440-021-00284-6 (PMC8460535; doi:10.1007/s43440-021-00284-6)
Supplement: Supplementary file 3 — Supplementary file3 (DOCX 518 KB) [file 43440_2021_284_MOESM3_ESM.docx]

**Supplementary material**

Novel Serotonin 5-HT_2A_ Receptor Antagonists Derived from 4-Phenylcyclohexane-5-Spiro-
and 5-Methyl-5-Phenylhydantoin, for Use as Potential Antiplatelet Agents

Anna Czopek^1^*, Monika Kubacka^2^, Adam Bucki^1^, Agata Siwek^3^, Barbara Filipek^2^, Maciej Pawłowski^1^, Marcin Kołaczkowski^1^

1 Department of Medicinal Chemistry, Jagiellonian University Medical College, 9 Medyczna Street, 30-688 Kraków, Poland

2 Department of Pharmacodynamics, Jagiellonian University Medical College, 9 Medyczna Street, 30-688 Kraków, Poland

3 Department of Pharmacobiology, Faculty of Pharmacy, Jagiellonian University Medical College, Medyczna 9, 30-688 Krakow, Poland


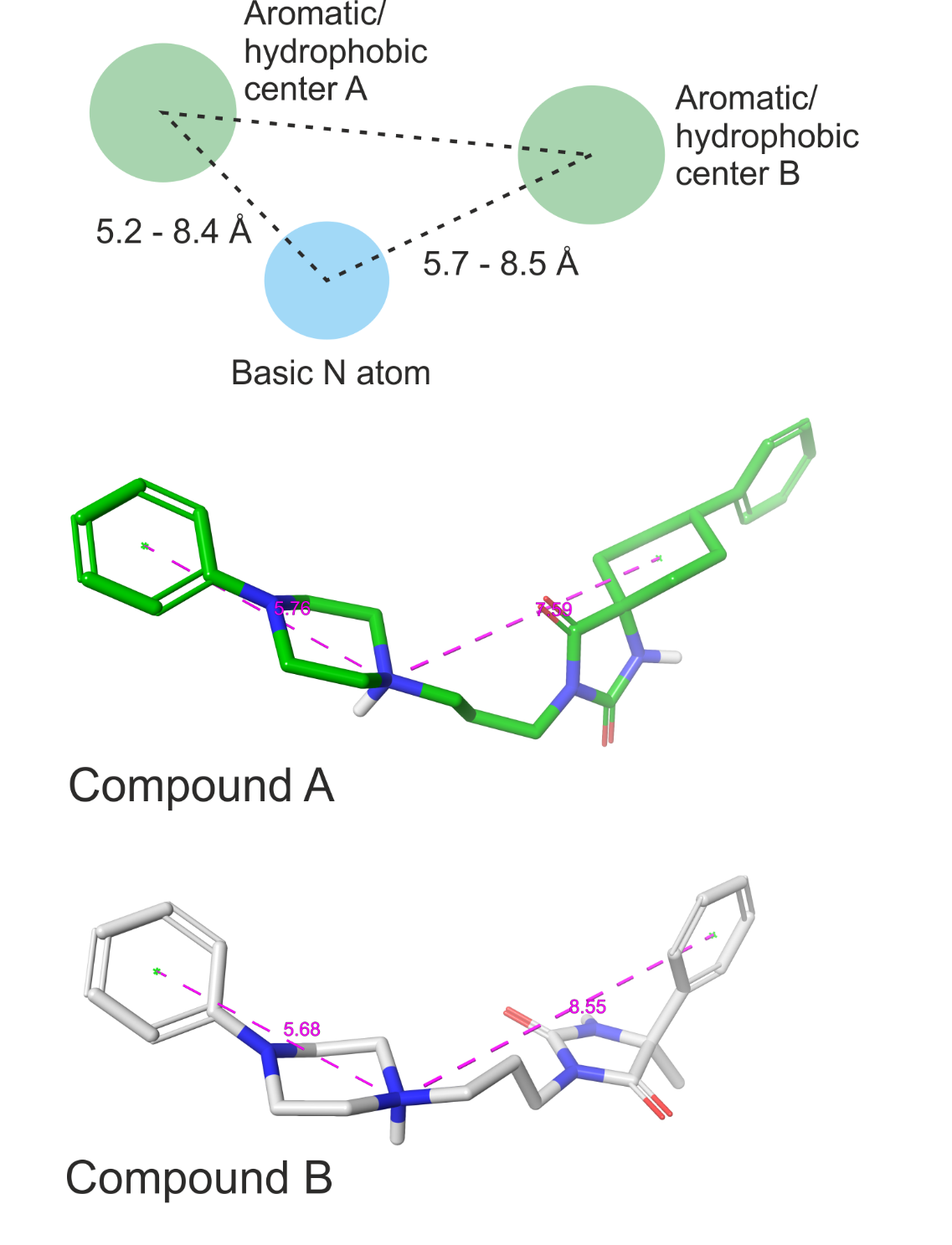


**Figure S1.** Common pharmacophore model for 5-HT_2A_ receptor antagonists [Urjita H Shah, Supriya A Gaitonde, José L Moreno, Richard A Glennon, Małgorzata Dukat, Javier González-Maeso, Revised Pharmacophore Model for 5-HT 2A Receptor Antagonists Derived from the Atypical Antipsychotic Agent Risperidone, ACS Chem Neurosci, 2019, 15;10(5):2318-2331. doi: 10.1021/acschemneuro] and distances between the basic amine and aromatic/hydrophobic rings of compounds A and B, which comply with requirements of the 5-HT_2A_ receptor pharmacophore model.


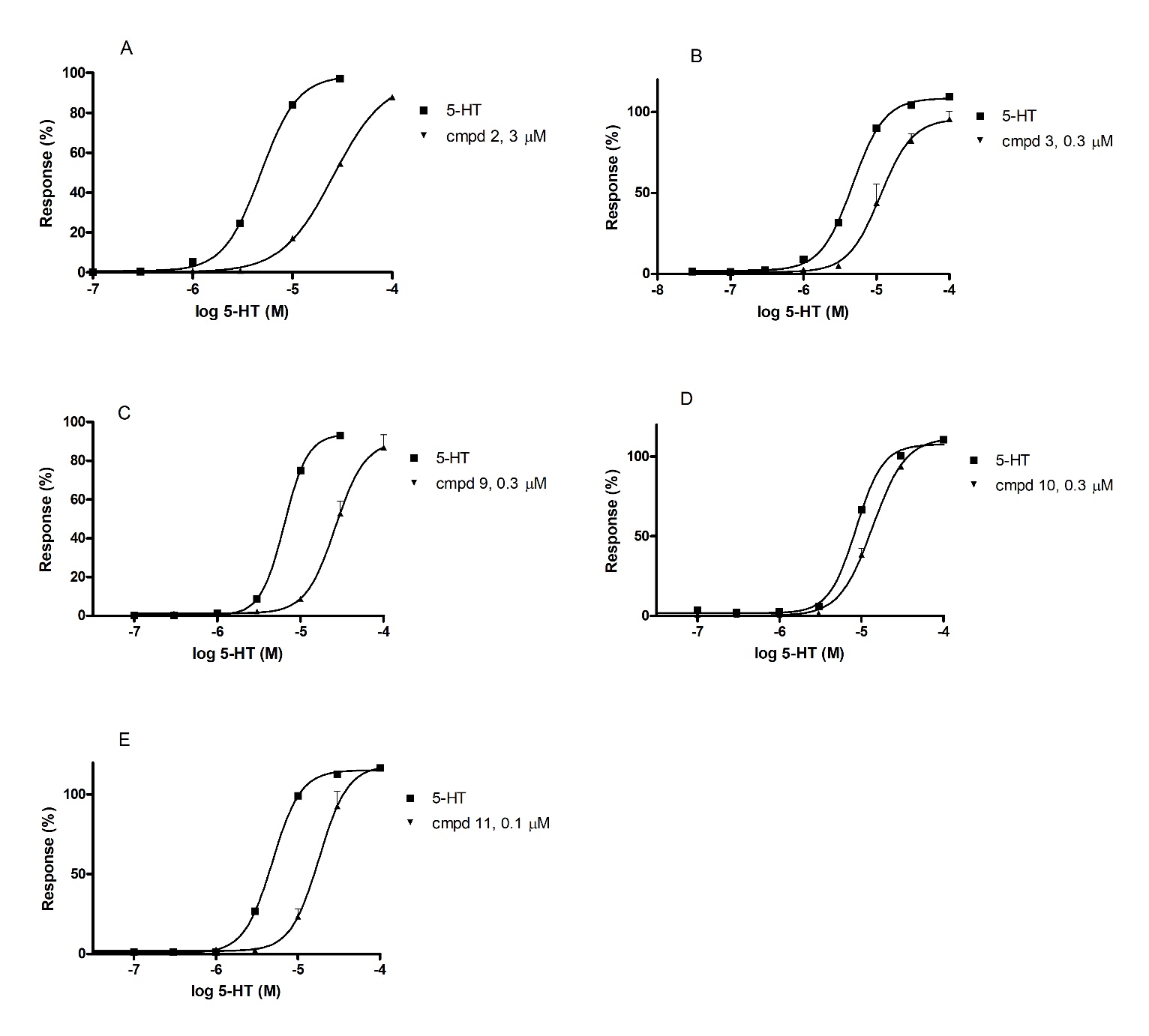


**Figure S2** Concentration-response curves to serotonin in the rat aorta in the absence (■) or presence of A) cmpd 2 (▼3 μM); B) cmpd 3 (▼0.3 μM); C) cmpd 9 (▼0.3 μM); D) cmpd 10 (▼0.3 μM); E) cmpd 11 (▼0.1 μM). Results are expressed as percentage of the maximal response to KCl depolarizing solution. Each point represents the mean ± S.E.M., (n = 3-4)

**Chemistry**

Description of compounds **1**, **6**, **7**, **8**, and **12**.

3-(2-(4-(4-Fluorobenzoyl)piperazin-1-yl)ethyl)-5-methyl-5-phenylimidazolidine-2,4-dione (1)

White powdery crystals. Yield: 34%; mp 117 – 119°C; TLC: *R_f_* = 0.16 (S_1_); HPLC: *t*_R_ = 1.083; MS calcd for [M + H]^+^: C_23_H_25_FN_4_O_3_ *m/z*: 424.19, found: 425.14; ^1^H NMR (300 MHz, CDCl_3_-*d*) δ ppm 1.83 (s, 3 H, -C*H_3_*) 2.43 (br. s., 4 H*,* Pip) 2.59 (t, *J*=6.15, 2.34 Hz, 2 H, -C*H_2_*-Pip) 3.22 (br. s., 2 H, Hyd-C*H_2_*-) 3.61 (t, *J*=6, 6.5, 7.6, 11.8 Hz, 4 H, Pip) 6.07 (s, 1 H, Hyd) 7.02 - 7.12 (m, 2 H, Ph) 7.30 - 7.42 (m, 5 H, Ph) 7.47 - 7.54 (m, 2 H, Ph). Anal. calcd for C_23_H_25_FN_4_O_3_ x 2H_2_O (460.51): C: 59.91, H: 6.35, N: 12.17; Found C: 60.08, H: 6.76, N: 12.03.

3-(3-(4-(4-Fluorobenzoyl)piperazin-1-yl)propyl)-5-methyl-5-phenylimidazolidine-2,4-dione (6)

White powdery crystals. Yield: 67%; mp 72 – 74°C; TLC: *R_f_* = 0.44 (S_2_); HPLC: *t*_R_ = 1.091; MS calcd for [M + H]^+^: C_24_H_27_FN_4_O_3_ *m/z*: 438.21, found: 439.23; ^1^H NMR (300 MHz, CDCl_3_-*d*) δ ppm 1.77 - 1.82 (m, 5 H, -C*H_3_,* -CH_2_-C*H_2_*-CH_2_-) 2.34 (t, *J*=7.03 Hz, 4 H, Pip) 3.36 (t, *J*=5.3, 8.2 Hz, 2 H, -C*H_2_*-Pip) 3.52 (t, *J*=1.8, 5.8, 7 Hz, 4 H, Pip) 3.68 (t, *J*=3.5, 5.3 Hz, 2 H, Hyd-C*H_2_*-) 6.32 (s, 1 H, Hyd) 7.03 - 7.12 (m, 2 H, Ph) 7.31 - 7.41 (m, 5 H, Ph) 7.45 - 7.51 (m, 2 H, Ph). Anal. calcd for C_24_H_27_FN_4_O_3_ (438.50): C: 65.74, H: 6.21, N: 12.78; Found C: 65.75, H: 6.41, N: 12.34.

3-(2-(4-(4-Fluorobenzoyl)piperazin-1-yl)ethyl)-8-phenyl-1,3-diazaspiro[4.5]decane-2,4-dione (7)

White powdery crystals. Yield: 86%; mp 224 – 226°C; TLC: *R_f_* = 0.28 (S_1_); HPLC: *t*_R_ = 1.382; MS calcd for [M + H]^+^: C_27_H_31_FN_4_O_3_ *m/z*: 478.24, found: 479.17; ^1^H NMR (300 MHz, CDCl_3_-*d*) δ ppm 1.66 - 1.82 (m, 4 H, Cyclohexane) 1.91 - 2.12 (m, 4 H, Cyclohexane) 2.49 (br. s., 4 H, Pip) 2.61 - 2.70 (m, 3 H, -C*H_2_*-Pip, Ph-C*H-*) 3.35 (br. s., 2 H, Hyd-C*H_2_*-) 3.61 - 3.74 (m, 4 H, Pip) 7.01 - 7.10 (m, 2 H, Ph) 7.17 - 7.25 (m, 1 H, Ph) 7.29 (d, *J*=4.10 Hz, 4 H, Ph) 7.33 - 7.40 (m, 2 H, Ph) 7.98 (s, 1 H, Hyd). Anal. calcd for C_27_H_31_FN_4_O_3_ x H_2_O (496.58): C: 65.31, H: 6.70, N: 11.28; Found C: 65.11, H: 6.83, N:11.01.

3-(2-(4-Benzhydrylpiperazin-1-yl)ethyl)-8-phenyl-1,3-diazaspiro[4.5]decane-2,4-dione (8)

White powdery crystals. Yield: 48%; mp 243 – 245°C; TLC: *R_f_* = 0.44 (S_1_); HPLC: *t*_R_ = 1.682; MS calcd for [M + H]^+^: C_33_H_38_N_4_O_2_ *m/z*: 522.30, found: 523.24; ^1^H NMR (300 MHz, CDCl_3_-*d*) δ ppm 1.66 - 1.82 (m, 4 H, Cyclohexane) 1.70 - 1.80 (m, 4 H, Cyclohexane) 1.88 - 2.04 (m, 4 H, Cyclohexane) 2.33 (t, *J*=1.00 Hz, 4 H, Pip) 2.51 (t, *J*=1.00 Hz, 4 H, Pip) 2.63 (t, *J*=6.45 Hz, 3 H, Ph-C*H*-, -C*H_2_*-Pip) 3.68 (t, *J*=6.45 Hz, 2 H, Hyd-C*H_2_*-), 4.18 (s, 1H, -C*H*-(Ph)_2_) 7.15 - 7.41 (m, 16 H, Ph, CDCl_3_) 8.45 (s, 1 H, Hyd). Anal. calcd for C_33_H_38_N_4_O_2_ (522.69): C: 75.83, H: 7.33, N: 10.72; Found C: 75.56, H: 7.44, N: 10.35.

3-(3-(4-(4-Fluorobenzoyl)piperazin-1-yl)propyl)-8-phenyl-1,3-diazaspiro[4.5]decane-2,4-dione (12)

White powdery crystals. Yield: 66%; mp 203 – 205°C; TLC: *R_f_* = 0.25 (S_3_); HPLC: *t*_R_ = 1.352; MS calcd for [M + H]^+^: C_28_H_33_FN_4_O_3_ *m/z*: 492.25, found: 493.26; ^1^H NMR (300 MHz, CDCl_3_-*d*) δ ppm 1.64 - 1.78 (m, 4 H, Cyclohexane) 1.85 (quin, J=7.18 Hz, 2 H, -CH_2_-C*H_2_*-CH_2_-) 1.94 - 2.12 (m, 4 H, Cyclohexane) 2.41 (t, J=7.03 Hz, 6 H, -C*H_2_*-Pip, Pip) 2.57 - 2.71 (m, 1 H, Ph-C*H-*) 3.41 (br. s., 2 H, Hyd-C*H_2_*-) 3.57 - 3.81 (m, 4 H, Pip) 7.08 (t, J=8.79 Hz, 2 H, Ph) 7.18 - 7.25 (m, 1 H, Ph) 7.28 - 7.33 (m, 4 H, Ph) 7.35 - 7.43 (m, 2 H, Ph) 7.71 (br. s., 1 H, Hyd). Anal. calcd for C_23_H_25_N_4_O_3_F (492.60): C: 68.27, H: 6.75, N: 11.37; Found C: 68.13, H: 6.90, N: 11.10.

5-HT_2A_ Receptor Binding Assay

Briefly: 50 µl working solution of the tested compounds, 50 µl [^3^H]-ketanserin (final concentration 1 nM) and 150 µl diluted membranes (5 µg protein per well) prepared in assay buffer (50 mM Tris, pH 7.4, 4 mM CaCl_2_, 0.1% ascorbic acid) were transferred to polypropylene 96-well microplate using 96-wells pipetting station Rainin Liquidator (MettlerToledo). Mianserin (10 μM) was used to define nonspecific binding. Microplate was covered with a sealing tape, mixed and incubated for 60 minutes at 27°C. The reaction was terminated by rapid filtration through GF/B filter mate presoaked with 0.5% polyethyleneimine for 30 minutes. Ten rapid washes with 200 µl 50 mM Tris buffer (4°C, pH 7.4) were performed using automated harvester system Harvester-96 MACH III FM (Tomtec). The filter mates were dried at 37°C in forced air fan incubator and then solid scintillator MeltiLex was melted on filter mates at 90°C for 5 minutes. Radioactivity was counted in MicroBeta2 scintillation counter (PerkinElmer).

*In vitro* functional bioassays at cells transfected with human 5HT_2A_ receptor (aequorin and luminescence-based intracellular calcium assay)

A cellular aequorin-based functional assay was performed with recombinant Chinese hamster ovary cells expressing mitochondrially targeted aequorin, human G-protein-coupled receptors and the promiscuous G protein α16 for 5-HT_2A_. After thawing, cells were transferred to assay buffer (Dulbecco's Modified Eagle Medium/HAM’s F12 with 0.1% protease free bovine serum albumin) and centrifuged. The cell pellet was resuspended in assay buffer and coelenterazine h was added at final concentrations of 5 μM. The cells suspension was incubated at 16°C, protected from light with constant agitation for 16 h and then diluted with assay buffer to the concentration of 120,000 cells/ml. After 1 h of incubation, 50 μl of the cells suspension was dispensed using automatic injectors built into the radiometric and luminescence plate counter MicroBeta2 LumiJET (PerkinElmer, USA) into white opaque 96-well microplates preloaded with test compounds. Immediate light emission generated following calcium mobilization was recorded for 30 s. In antagonist mode, after 30 min of incubation the reference agonist was added to the above assay mix and light emission was recorded again. Final concentration of the reference agonist was equal to EC_80_ of α-methylserotonin (30 nM).

**Table S1.** Binding affinity of compounds **13** and **14** for serotonin 5-HT_1A_, 5-HT_2A_, and dopamine D_2_ receptors.

| **Compd** | **K_i_ ± SEM [nM]*** | | |
| --- | --- | --- | --- |
|  | **5-HT_1A_** | **5-HT_2A_** | **D_2_** |
| **13** | 1668 ± 75 | 20 ± 2 | >10000 |
| **14** | 5211 ± 268 | 56 ± 7 | >10000 |

*Data from [A. Czopek, A. Zagorska, M. Kolaczkowski, A. Bucki, B. Gryzlo, J. Rychtyk, M. Pawlowsk, A. Siwek, G. Satala, A. Bojarski, M. Kubacka, B. Filipek, New Spirohydanoin Derivatives - Synthesis, Pharmacological Evaluation, and Molecular Modeling Study., Acta Pol. Pharm. 73 (2016) 1545–1554.]

**Table S2.** In silico toxicity prediction values for the most active compounds **2-5, 9-11, 13** and **14**.

| **Toxycity model name** | **Ketanserin** | **Compd 2** | **Compd 3** | **Compd 4** | **Compd 5** | **Compd 9** | **Compd 10** | **Compd 11** | **Compd 13** | **Compd 14** |
| --- | --- | --- | --- | --- | --- | --- | --- | --- | --- | --- |
| **AMES toxicity** | No | No | No | No | No | No | No | No | No | No |
| **Max. tolerated dose (human) [log mg/kg/day]** | 0.192 | 0.496 | -0.554 | -0.613 | -0.641 | -0.543 | -0.597 | -0.576 | -0.494 | -0.511 |
| **hERG I inhibitor** | No | No | No | No | No | No | No | No | No | No |
| **hERG II inhibitor** | Yes | Yes | Yes | Yes | Yes | Yes | Yes | Yes | Yes | Yes |
| **Oral Rat Acute Toxicity (LD50) [mol/kg]** | 2.333 | 2.323 | 2.519 | 2.576 | 2.593 | 2.981 | 3.094 | 3.114 | 2.959 | 3.033 |
| **Oral Rat Chronic Toxicity (LOAEL)**  **[log mg/kg_bw/day]** | 1.177 | 1.623 | 1.384 | 1.375 | 1.361 | 0.73 | 0.86 | 0.728 | 0.765 | 0.827 |
| **Hepatotoxicity** | Yes | No | Yes | Yes | Yes | Yes | No | No | Yes | No |
| **Skin Sensitisation** | No | No | No | No | No | No | No | No | No | No |
| ***T.Pyriformis* toxicity**  **[log ug/L]** | 0.291 | 0.294 | 0.759 | 0.776 | 0.748 | 0.528 | 0.538 | 0.523 | 0.571 | 0.557 |
| **Minnow toxicity**  **[log mM]** | 2.453 | 5.741 | 2.807 | 2.731 | 2.818 | 5.264 | 5.055 | 5.12 | 5.147 | 4.614 |
